# Effects of Maternal Deprivation on Anxiety, Depression, and Empathy in Male and Female Offspring of Wistar Rats in the Face of Novel Objects

**DOI:** 10.31661/gmj.v0i0.1093

**Published:** 2019-01-01

**Authors:** Solmaz Khalifeh, Fariba Khodagholi, Mehrad Moghtadaei, Ali Behvarmanesh, Afshin Kheradmand, Hamed Ghazvini

**Affiliations:** ^1^Cognitive and Neuroscience Research Center (CNRC), Tehran Medical Sciences, Islamic Azad University, Tehran, Iran; ^2^Neuroscience Research Center, Shahid Beheshti University of Medical Sciences, Tehran, Iran; ^3^Department of Physiology, Faculty of Advanced Sciences and Technology, Tehran Medical Sciences, Islamic Azad University, Tehran, Iran; ^4^AmirAlmomenin Hospital, Tehran Medical Sciences, Islamic Azad University, Tehran, Iran; ^5^Departmant of Pharmacology and Toxicology, school of Pharmacy, International campus, Iran University of medical science,Tehran,Iran; ^6^Psychiatry and Behavioral Sciences Research Center, Addiction Institute, Mazandaran University of Medical Sciences, Sari, Mazandaran, Iran

**Keywords:** Maternal Deprivation, Empathy, Anxiety, Depression, Wistar Rat

## Abstract

**Background:**

Early life stress (ELS) models such as maternal deprivation (MD) are used to investigate behavioral changes in rodents under stressful situations. MD is a situation in which rat pups are separated from the dam; MD has different paradigms. The purpose of this research is to evaluate the effects of maternal deprivation on anxiety, depression, and empathy in adult Wistar rats.

**Materials and Methods:**

MD was applied to pups as per specifically designed protocol to compare rats of the control group with maternal deprivation rats and also the group, which faced novel objects. Each group consisted of eight rats. In this study, separation started from postnatal day (PND) 14 for various periods up to PND 60. EPM test was undertaken to measure anxiety; moreover, FST was used to indicate levels of depression. Also, changes in the empathy ratio were also demonstrated. One-way analysis of variance (ANOVA), Tukey’s post hoc analysis, and t-test were applied to analyze the results.

**Results:**

MD-treated rats showed a significant decrease in anxiety and empathy indexes compared with those in the control group (P<0.05). However, MD significantly increased depression in both male and female rats (P<0.05). Finally, exposure to novel objects decreased depression but did not have any effect on anxiety and empathy levels in MD rats (P<0.05).

**Conclusion:**

ELS may lead to various states of mood and behavior in adulthood. According to the findings of this study, depression increases due to MD, though both anxiety and empathy decrease in both male and female Wistar rats. Moreover, exposure to novel objects decreases depression, while anxiety and empathy do not change significantly with exposure to novel objects.

## Introduction

In order to investigate behavioral reactions in rodents under stressful situations, scientists have used early life stress (ELS) models such as maternal deprivation (MD) and early weaning. MD is used to separate rat pups from the dam and it has different paradigms Behavioral reactions in adolescence and expansion of brain in mammals is badly affected by examples of ELS such as MD [1]. The existence of some barriers in methodology has prevented appropriate studies on the effects of MD on early PND development of rodents, and possible cognitive changes in adult individuals [2]. One of the possible psychiatric disorders emergent from MD is anxiety [3-5]. In this study, the effect of MD on anxiety in Wistar rats in the presence and absence of novel objects is studied. The second psychiatric disorder studied in this study is depression, which is a major concern for public health. The World Health Organization (WHO) predicts that the majority of human beings will be challenged by depression by 2020 [6]. Depression is a frequent, chronic, recurrent, and life-debilitating disorder that leads to morbidity and mortality. Depression has a complex structure in humans and affects the development of animal models [7]. Rodents are among the most useful species for studying social behavior. They manifest emotional contagion and cooperation and have pro-social behaviors. These last include some actions that lead to increase in the well-being of others: such actions are found widely in animals, as being pro-social in a group promotes individual reproduction and survival [8-11]. Animal groups usually vie for valuable resources, which are limited. Thus, it seems that pro-social behaviors are not adaptive toward other group’s members. It can be understood that the social context plays a major role in the animal’s motivation to act pro-socially or aggressively [12-15]. Finally, this research reveals the effect of MD on empathy in Wistar rats’ offspring, as a pro-social behavior, in the presence and absence of novel objects.

## Materials and Methods

### 1. Animals

In the present experiments, adult male and female albino Wistar rats (Pasteur Institute, Tehran, Iran) weighing from 200 g to a maximum of 250 g were put together in standard cages (four/cage) in a temperature controlled room (20–22 °C) on a standard 12h light/dark cycle with lights on from 07:00 a.m. to 19:00 p.m.. Animals had free access to food and drinking water. In these tests, both female and male rats were used for behavioral evaluations. Testing was started sixty days after birth, at which time animals were weighed, cages were cleaned, and general health status was checked. All experiments were done according to the Guide for the Care and Use of Laboratory Animals (National Institutes of Health Publication No. 80–23, revised 1996) and were confirmed by the Research Committee of the Shahid Beheshti University of Medical Sciences.

### 2. Experimental Design

At first, females rats were mated by males. Female rats stayed together for another two weeks and were individually housed to prepare for birth. A paper towel was provided for each mother as nesting material. After the delivery of pups, their sex was determined. The day of delivery was denoted as postnatal day (PND). At PND 15, litters were randomly divided into have been mated by groups and specified for the MD and control condition. Litters of the control group were placed back into their home cage while litters of the experimental group remained together: MD took place on the latter. Between PND 15 to PND 22, the litters were removed from their dams and were put in a separate cage in the adjacent room. Litters remained together in another cage for 10 min (from 10 a.m. to 14 p.m.) every day. From PND 23 to 29, 5 min was added to the separation time each day (day 23: MD 15 min; day 29: MD 45 min). From PND 30 to PND 60 (testing day), the dam was separated from litters for 1 hour every day. At PND 30, litters of the experimental group were classified into two groups with two subgroups according to their sex. In the first group, normal MD took place on the litters, while female and male litters were separated from each other in separate cages. In the second group, normal MD took place on litters in the presence of the novel object. While female and male litters were separated from each other in separate cages, litters of this group faced two new novel objects each day, with a specific protocol‒this protocol was an experimental design. On the first day (PND 30), litters faced with two novel objects (object 1 and object 2). On the second day, a new object (object 3) was added to the others. On the third day, a new object (object 4) was added to the last three objects. On the fourth day, a new object (object 5) was added even as object 1 and 2 were removed. This process continued until the testing day (PND 60). Data from normal rats, which had seen novel objects every day were the same as control groups; to avoid repetition we merged this group as a control group.

### 3. Behavioral Tests

#### 3.1. Elevated Plus-Maze Test (EPM)

There is a wide range of tests measuring anxiety levels; EPM test is one of them. EPM is based on the conflict between the exploration instinct of rodents facing new places versus the fear of standing in open and elevated arms [16]. The maze was either wooden or iron, consisting of two closed alleys (50 cm ×10 cm ×40cm) and two open alleys (50 cm ×10 cm). The alleys were linked by a central 10 x 10 cm area. The maze was cleaned by water and neutral detergent and dried each time. The habituation took place before the test. Litters were located in the central part of the maze. They were picked up and put back into their cages after 5 min — this procedure aimed to increase the likelihood of entering open arms and optimizing the test’s sensitivity. Firstly, the rat was placed in the center of the plus maze. Its head faced an open arm, ensuring that it remained free to explore the apparatus for 5 min [17]. Percentage of time spent in open arms (%OAT: [time in open arm/time in “open + closed” arm] ×100) and percentage of open arm entries (%OAE: [number of open arm entries/number of “open + closed” arm entries] ×100) were considered as an index of anxiety. Locomotor activity was indicated by the number of total entries [18].

#### 3.2. Forced Swiming Test (FST)

The habituation procedure took place at least 30 min before the beginning of the test. Then, each rat was gently placed into a cylindrical glass swim tank (45 cm in hight and 20 cm in diameter) for 7 min. The tank was filled with water (24 ± 1°C) to a depth of 30 cm [19], thus ensuring that the rat had nobody motion except the small necessary ones to keep itself alive. This was defined as immobility time and was considered a marker of depression [20]. On the other hand, pedaling and making circular movements by a rat is considered as swimming behavior. The water was changed after each trial to eradicate the smell left from the previous experiment. Finally, the animals were caught from the water and put back to their cages. Paper towels were used to drying the rats [21].

#### 3.3. Measurment of Empathy

In accordance with research on rodents’ emotional contagion to determine the capacity of rats’ empathy-based motivation towards helping behavior, the following method was used to find out whether or not the presence of a trapped rat from the same sex promotes a pro-social motivational state in experimental rats and leads them to set free the rat captive in the restrainer. In each session, a rat (the free rat) was placed in an area within a centrally located restrainer in which another rat was trapped for 5 min. The trapped rat and the free one had the same strain, but the trapped rat was unrecognized for the free rat. The free rat circled the restrainer furthermore, digging and biting it, and contacted the trapped rat through holes in the restrainer. The movement time of the free rat around the restrainer was measured: the results determined the empathy level in the free rat [22].

### 4. Statistical Analysis

Each experiment was repeated at least three times. Mean ± SEM (standard error of the mean) was used to express the data, which was processed by Graph Pad Prism® 5.0. United States of America, One-way analysis of variance (ANOVA), post hoc analysis Tukey’s, and t-test were used. A p-value less than 0.05 was considered statistically significant.

## Results

### Effects of MD On Anxiety in Rats

**Figure 1:**
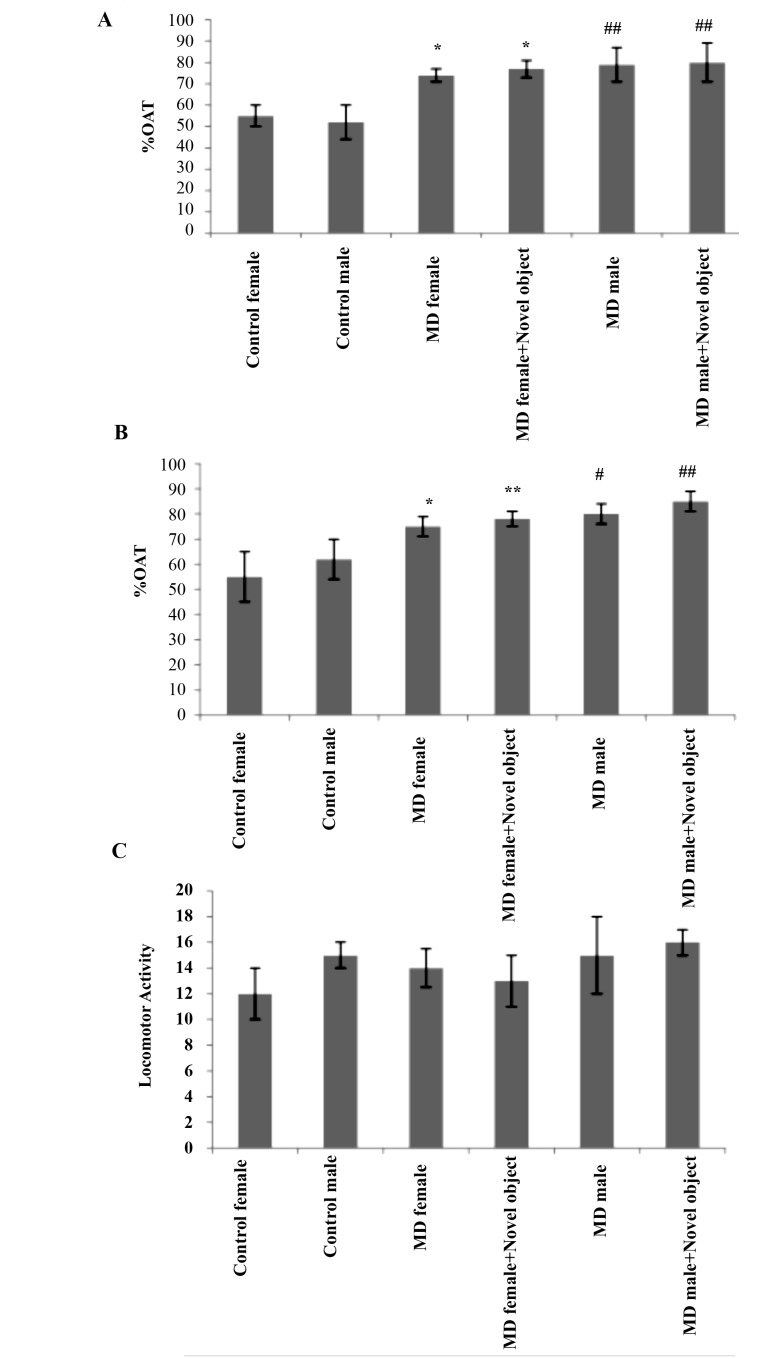
Effect of MD on anxiety that indicated by a change in %OAT (A), %OAE (B), and locomotor activity(C) and also the effect of exposure to novel objects in male and female rats (n=8). The symbol * shows remarkable differences in comparison to the control female group, and the symbol # shows significant differences in comparison to the control male animals. * and # P<0.05, ** and ## P<0.01

According to Figure-1, both male and female rats show significant difference regarding anxiety behavior using EPM in comparison with the control groups. According to these results, MD increases significantly with exploration time in the open alleys in both female and male rats (P <0.05 and P<0.01, respectably). This indicates reduced anxiety-like behavior in them. The results also demonstrated that the existence of novel objects increased the %OAT in MD encounter rats similar to MD groups as compared to control animals (P<0.05 and P<0.01 in female and male MD groups, respectably, both of which saw novel objects). Furthermore, the percentage of entries in open arm (%OAE) also showed a significant increase among both male and female MD treated rats (P<0.05 in both of them compared to control rats) and rats with the experience of facing novel objects (P<0.01 in male and female rats compared to control rats). However, there was no significant change in locomotor activity in experimental groups.

### Effects of MD On Depression in Rats

**Figure 2:**
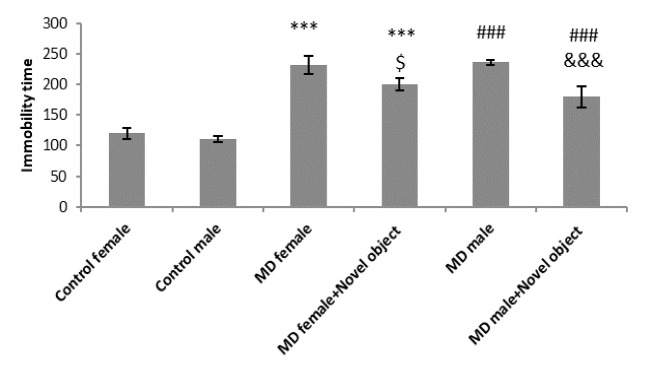
Effect of MD on depression (indicated by a change in immobility time) and also the effect of exposure to novel objects on depression in MD treated male and female rats (n=8). The symbol * shows remarkable differences in comparison to the control female group, and the symbol # shows significant differences in comparison with the control male rats. Symbol $ shows remarkable differences in comparison to the MD female group, and the symbol & shows remarkable differences in comparison to the MD male group. * and # P<0.05, *** and ### P<0.001

As the original definition explained above, the depression level of the rodents was measured by FST. Immobility time was taken as the index of depression. In other words, higher immobility time means higher depressive state while lower immobility time shows lower depressive state. The results demonstrated that MD significantly increased immobility time in both male and female rats (P<0.001). These results were also observed in male and female MD treated rats that faced novel objects compared to controls (P<0.001). Being exposed to novel objects decreased immobility time in both female and male MD rats (P<0.05 and P<0.001, respectably) in comparison to MD groups (Figure-2).

### Effects of MD On Empathy in Rats

**Figure 3:**
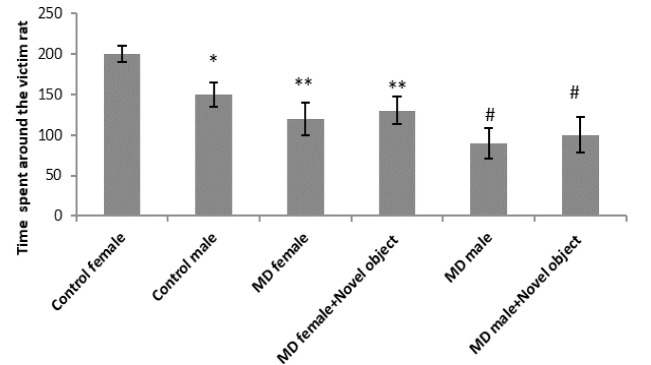
Effect of MD on empathy (indicated by a change in time spent around the victim rat) and also the effect of exposure to novel objects on empathy in MD treated male and female rats (n=8). The symbol * shows remarkable differences in comparison with the control female group and the symbol # shows remarkable differences in comparison with the control male animals. * and # P<0.05, ** and ## P<0.01

In this test, the aim was to observe empathy in rats by measuring the time they spent around victim rats. A significant decrease was seen in the time spent around victim rats in both male (P<0.05, Figure-3) and female rats treated with MD (P<0.01). Results of male and female rats treated with MD who faced novel objects were the same as MD rats.

## Discussion

In this study, we demonstrated the effects of MD on anxiety, depression, and empathy in Wistar rats. Another aim of this study was to indicate the results of exposure to novel objects in MD treated rats: i.e., to observe changes in anxiety-like behaviors, depression, and empathy in rats. In depression and anxiety tests, the presence of novel objects had a significant effect on the behaviors of rats. EPM allowed prediction of anxiogenic- or anxiolytic-like effects of drugs or treatments in rodents [23]. Previous studies on the effects of MD on anxiety demonstrated different results. According to a recent survey, MD decreased anxiety. There are some differences between their study and this research: these are the protocol of MD, the strain of rats, and tests. They used the open field test to determine change, and the strain of their rats was Fischer and Lewis. Regarding their MD protocol, between PND 1 and 28, MD rats were separated from their dams for 2h per day. The mother was withdrawn from the cage and was put in a separate cage aside the home cage, while the pups remained in their nest cage. For each mother, a corresponding cage was used during the tail [24]. There is another study that reported that dendritic length decreased in MD rats in comparison to rats in the control group. This result led to an increase in anxiety-like behaviors. Their animals were Sprague-Dawley rats, and they used a different MD protocol in comparison with our experimental design. In their study, maternal separation pups were separated every day from their dams for 3h from PND 6 to 21 [25]. The results of the present study indicate that MD can produce an anxiolytic-like effect in both male and female rats. The anxiolytic-like effect of MD is associated with an increase in %OAT and %OAE. Remaining in the open alleys meant lower levels of anxiety: results show that MD decreased the level of anxiety in both male and female MD treated rats. Also, the results show that the presence of novel objects has a significant effect on %OAT and %OAE. Increase in OAE is also shown that the presence of novel objects reduces anxiety. Depression is known as a complex psychological disorder containing symptoms such as low mood, feeling sad, anhedonia, helplessness, and a feeling of worthlessness or shame. It is impossible to detect and quantify such feelings in rodents. Researchers have used FST in the absence of direct methods for diagnosing depression in rodents [26]. In FST, a rat experiences an inescapable forced swim and is tested for “learned helplessness,” described by floating and fewer attempt to swim or climb on the walls of the cylinder [27]. There is a recent study about the effect of MD on depression in Long-Evans rats. Results demonstrate that MD leads to depression-like behavior only in female rats. According to their findings, swimming time increases remarkably in female rats subjected to FST. This indicates a decrease in depression. However, male rats did not report change in depression [28]. According to the results of this research, MD increased the duration of floating and decreased swimming and/or climbing. Increasing immobility time means a high level of depression. The result is that MD significantly induces depression in both male and female rats. Seeing novel objects decreased immobility time and depression in both male and female rats treated with MD as compared to MD rats without exposure to a novel object. Empathy is the capacity to recognize and share another’s emotions. In human beings, empathy sometimes motivates pro-social behavior and caring [29, 30]. Some groups of empathy are mostly related to subcortical neural structures, which remain unchanged among different mammalian species [31]. The emotional contagion that has been detected in rodents is a basic part of empathy eventuating when the emotional state of one promotes a similarly linked emotion in another. Thus, mammals and rodents may have a shared mechanism for mobilizing pro-social stimulants in reaction to the confusion of others in their species [32-35]. Our findings demonstrate that no other study reports the effect of MD on empathy. Given the results of this study, MD seems to have a remarkable influence on the empathy of rats: it significantly decreased the time spent around victim rats in both male and female rats and resulted in decrease in empathy. This means that MD treated rats did not attempt to liberate the trapped rat as much as the control rats. Our data demonstrate that exposure to novel objects increased empathy for MD treated male and female rats. Our research shows that helping another rat by trying to liberate it from a restrainer is obviously related to previous social experiences [9, 29, 30, 36, 37] According to the studies, the type of empathy in rats is the principal inspiration for their helping behavior [35, 38-43]

## Conclusion

Experiencing stressful situations in early life could have different effects on mood and behavior in adulthood. According to the results obtained in this study, MD decreased anxiety and empathy but increased depression in both male and female rats. Exposure of novel objects decreased depression but increased empathy and anxiety in MD rats as compared to the MD groups, which were not exposed to novel objects.

## Acknowledgment

We are grateful to Prof. Mohammad-Reza Zarrindast for providing advice and valuable remarks.

## Conflict of Interest

The authors declare that they have no conflict of interest.
